# Support Enzyme Loading Influences the Effect of Aldehyde Dextran Modification on the Specificity of Immobilized Ficin for Large Proteins

**DOI:** 10.3390/molecules29153674

**Published:** 2024-08-02

**Authors:** El Hocine Siar, Pedro Abellanas-Perez, Javier Rocha-Martin, Roberto Fernandez-Lafuente

**Affiliations:** 1 Departamento de Biocatálisis, ICP-CSIC, Campus UAM-CSIC, 28049 Madrid, Spain; hocines1@hotmail.fr (E.H.S.); m.a.p@icp.csic.es (P.A.-P.); 2 Agri-Food Engineering Laboratory (GENIAAL), Institute of Food, Nutrition and Agri-Food Technologies (INATAA), University of Brothers Mentouri Constantine 1, Constantine 25017, Algeria; 3 Department of Biochemistry and Molecular Biology, Faculty of Biology, Complutense University of Madrid, José Antonio Novais 12, 28040 Madrid, Spain

**Keywords:** steric hindrances, size specificity, tuning enzyme specificity, enzyme loading, immobilized proteases

## Abstract

It has been reported that the modification of immobilized glyoxyl–ficin with aldehyde dextran can promote steric hindrances that greatly reduce the activity of the immobilized protease against hemoglobin, while the protease still maintained a reasonable level of activity against casein. In this paper, we studied if this effect may be different depending on the amount of ficin loaded on the support. For this purpose, both the moderately loaded and the overloaded glyoxyl–ficin biocatalysts were prepared and modified with aldehyde dextran. While the moderately loaded biocatalyst had a significantly reduced activity, mainly against hemoglobin, the activity of the overloaded biocatalyst was almost maintained. This suggests that aldehyde dextran was able to modify areas of the moderately loaded enzyme that were not available when the enzyme was overloaded. This modification promoted a significant increase in biocatalyst stability for both biocatalysts, but the stability was higher for the overloaded biocatalyst (perhaps due to a combination of inter- and intramolecular crosslinking).

## 1. Introduction

Proteases have multiple applications as industrial biocatalysts [1,2,3,4,5,6,7,8,9,10], including the production of peptide bonds [9,11,12,13], the production of bioactive peptides via the selective hydrolysis of proteins [14,15,16,17], applications in the detergent [18,19,20] and dairy [21,22,23,24] industries, the tenderization of meats [25,26,27], aquaculture applications [28,29,30], the modification of fermented products [30], antibacterial applications, biofilm prevention [31,32,33,34,35], applications in the textile industry [36,37,38,39,40] and applications as therapeutic agents [38,41,42,43,44,45]. With this wide range of applications, an interest in the ability to tune protease features becomes evident.

Enzyme immobilization has been recognized as a strategy that can greatly improve enzyme features [46,47,48,49,50,51,52,53,54]. In many applications, the use of immobilized proteases may be advantageous, and even fully necessary in some cases (even with the existence of some specific problems that protease immobilization may create in the production of an active biocatalyst) [2,30,55]. Immobilization prevents the contamination of the product by the protease, permitting stricter control of the reaction (the reaction should stop after biocatalyst removal) and preventing any negative effect that the presence of the enzyme in the final product may have on the consumer (e.g., allergic reactions) [56,57,58,59]. Protease stabilization using proper immobilization protocols (e.g., via multipoint covalent attachment) may increase enzyme stability and thus increase the range of conditions in which the enzyme may be utilized [48,60,61,62,63,64,65]. It has been reported that the use of a variety of immobilization protocols can also alter enzyme features (e.g., selectivity, specificity and inhibition) [66,67,68,69,70,71,72,73,74,75,76,77,78,79,80,81,82]. 

One recently reported possibility of using immobilization to tune protease properties is to increase the protease’s preference for small proteins versus large proteins using the controlled generation of tailor-made steric hindrances, which would hinder the ability of the immobilized enzyme to accommodate large proteins in its active site [83,84]. For this purpose, in these research efforts, a layer of inert protein was co-immobilized over a layer of protease that fully coated the support surface [84], while in another instance the protease was co-immobilized with a larger protein on the support surface (e.g., hemoglobin) [83]. In another example, the immobilized protease was modified with aldehyde dextran [83]. This latter case is another successful example in which the chemical modification of the previously immobilized/stabilized enzymes may improve the properties of these enzymes [85]; aldehyde dextran was used in this case [86] (together with the stabilization of multimeric enzymes by crosslinking all enzyme subunits or the generation of hydrophilic microenvironments) [87,88,89,90]. 

**Figure 1:**
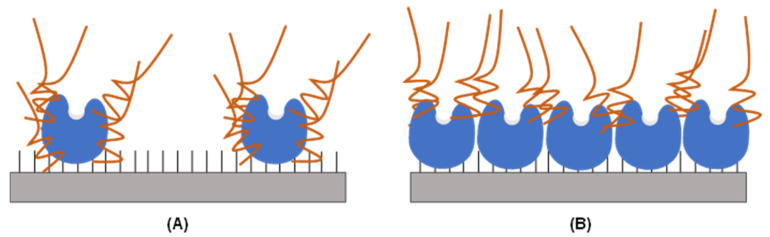
Schematic representation of the aldehyde dextran modification of moderately loaded (**A**) and overloaded (**B**) immobilized enzyme biocatalysts.

That way, the modification of the immobilized enzyme with aldehyde dextran can decrease the enzyme’s activity against large proteins. However, this modification can also have some other positive effects on enzyme features (e.g., stability), and if the purpose of the biocatalysts is to fully hydrolyze large and small proteins, this change in enzyme specificity regarding protein size may become disadvantageous. It is also possible that both immobilization and chemical modification can affect the specificity of the ficin extract for different proteins; this cannot be discarded as an explanation of the reported results. However, it is likely that the coating of the protein surface with aldehyde dextran can have a different effect depending on the loading of the support. If a small amount of enzyme is loaded onto the support surface, then the dextran modification can affect the entire portion of the enzyme surface that is not in contact with the support surface (Figure 1). However, if the support surface is full of enzyme molecules, then it can be expected that only the areas of the enzyme farthest from the support surface can be modified by steric reasons. That way, tese modifications with polymers can have different effects on enzyme properties depending on the loading of the support (Figure 1).

In this paper, we used ficin as a model enzyme [91,92]. This enzyme is a thiol protease, and it has multiple uses [91], including in the cheese industry [93,94], the proteolysis of proteins [95,96,97,98,99,100], the prevention of microbial biofilms [101,102], the production of miniature antibodies [103,104] and the synthesis of peptides [105,106,107,108].

We have previously immobilized this enzyme on glyoxyl agarose, and this immobilized ficin biocatalyst exhibited an improved stability [109], although the oxidation of its catalytic Cys residues reduced the potential of this technique to increase enzyme stability in the presence of oxygen [110]. This immobilized enzyme maintains its proteolytic activity [109] and can be used to hydrolyze proteins or even to produce milk coagulum [111]. Our objective in this new research effort is to analyze if the effect of the modification with dextran aldehyde on the size specificity of the immobilized ficin depends on the loading of the biocatalyst using two very different support enzyme loadings. These loadings are based on previous results from our group [111]. We utilized 10 mg of ficin per g of support (around 15–20% of maximal loading) and 90 mg of ficin extract per g of support, a loading amount that is almost within the limit of the capacity of the support, thus producing biocatalysts with better properties regarding milk clogging. 

## 2. Results and Discussion

### 2.1. Preparation of Modified Glyoxyl–Ficin Agarose Biocatalysts

**Figure 2:**
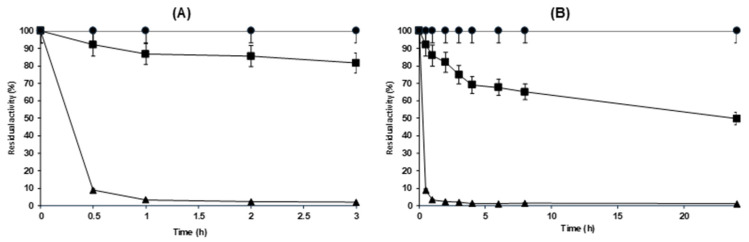
Immobilization courses of ficin on glyoxyl agarose supports at 1 mg/mL (**A**) and 9 mg/mL (**B**). These immobilizations were carried out at pH 10.05 and 25 °C using 1 g of support per 10 mL of suspension. Additional information is available in the Materials and Methods section. Squares: suspensions; triangles: supernatants; circles: free enzymes.

Figure 2 shows the immobilization course of ficin on glyoxyl agarose with an enzyme loading exceeding the support capacity [111], and also with moderate loading. With moderate enzyme loading, all enzymes were immobilized in 1 h and maintained 80% of their initial activity. With an enzyme loading that exceeded the capacity of the support [111], 10% of the activity remained in the supernatant even after 24 h, and the expressed activity was around 60%. This lower activity was expected due to an increase in the importance of substrate diffusion matters.

**Figure 3:**
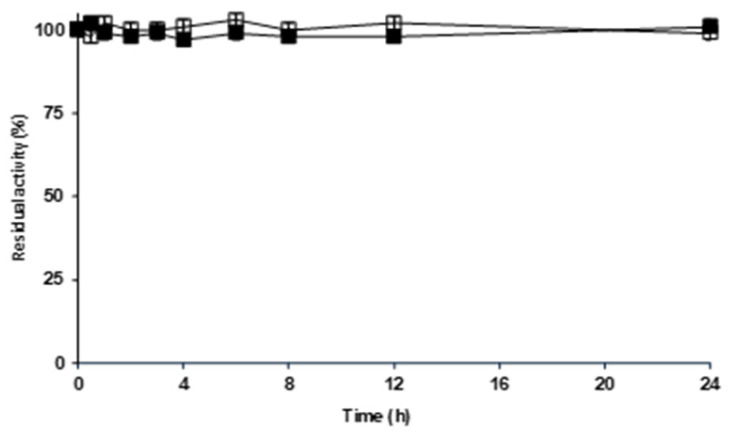
Effect of the aldehyde dextran modification (pH 7 and 25 °C) on the activities of overloaded and moderately loaded ficin biocatalysts. Other specifications may be found in the Materials and Methods section. Full squares: glyoxyl–ficin at 1 mg/mL; empty squares: glyoxyl–ficin at 9 mg/mL.

Figure 3 shows the effect of the incubation of both glyoxyl–ficin biocatalysts with aldehyde dextran on ficin activity versus BAPNA. The activity did not decrease even after 24 h of aldehyde dextran modification of the enzyme, suggesting that the modification did not have a significant effect on the enzyme structure or its active site. The reaction with the Schiff reagent before the reduction step confirmed the presence of aldehyde dextran on the biocatalysts, while the reduced biocatalysts did not produce any color, which was the case for the naked and the reduced glyoxyl support after incubation with aldehyde dextran and further washing. Therefore, aldehyde dextran was attached to the immobilized ficin molecules. Free ficin had 70% of the activity using hemoglobin compared to when casein was used. The proteolytic activity of the overloaded ficin preparation was better retained against casein (65%) than it was against hemoglobin (40%), while the opposite was observed using the moderately loaded biocatalysts (55% using casein and 66% using hemoglobin). This interesting change in substrate specificity, although not very large, may warrant further investigation.

### 2.2. Effect of the Dextran Modification on the Protein Substrate Size Specificity of Immobilized Ficin 

Next, the proteolytic activity of the immobilized ficin was compared to that of the immobilized, aldehyde dextran-modified ficin (Table 1). With casein as the substrate, which is a protein that is similar in size to ficin, the moderately loaded biocatalyst had a 30% decreased activity at 37 °C and a 20% decreased activity at 55 °C, while the overloaded biocatalysts had a decrease in activity of just over 5% at 37 °C, and the modification had no relevant effect at 55 °C. This decrease may be explained by the generation of steric hindrances that hinder the ability of the immobilized ficin molecules to access casein or changes in enzyme specificity caused by the chemical modification of the immobilized ficin extract [83]. The effect was greater at lower temperatures than at higher ones, as the increase in temperature may have increased the mobility of the dextran layer and the casein molecule. However, this relatively small protein (casein) was still able to access the active site of the immobilized and modified ficin, and the ficin specificity was maintained. The smaller effect of the modification on the overloaded biocatalyst was evident compared to the moderately loaded one. This may be explained by lower loading, as the external area of the enzyme is susceptible to modification by dextran, which can result in a greater effect on the proteolytic activity.

These effects were maximized when hemoglobin, a large protein, was used as the substrate. The activity of the moderately loaded biocatalyst at 55 °C slightly exceeded 10% of that of the unmodified biocatalyst, while at 37 °C, the remaining activity was under 10%. However, the activity of the overloaded biocatalyst was over 90% at 55 °C and slightly under 90% at 37 °C. This confirmed the trend observed with casein, but the effect was much greater. Of note, this change may have occurred due to a modification-induced alteration in the protein specificity of the immobilized enzyme that reduced its ability to hydrolyze hemoglobin.

**Figure 4:**
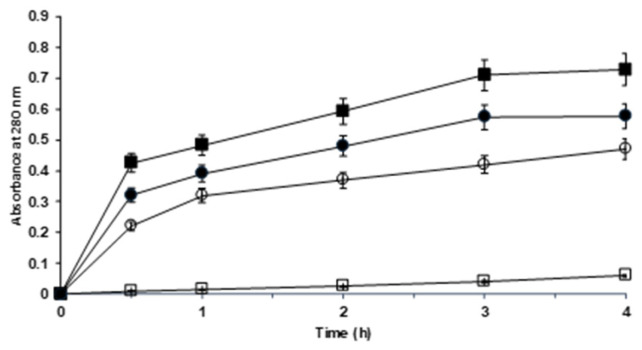
Hydrolysis courses of hemoglobin with the different glyoxyl–ficin biocatalysts. Experiments were carried out at 37 °C and pH of 7. For further details, check the Materials and Methods section. Full squares: moderately loaded glyoxyl–ficin; empty squares: moderately loaded glyoxyl–ficin–dextran at 1 mg/mL; full circles: overloaded glyoxyl–ficin; empty circles: overloaded glyoxyl–ficin–dextran.

To validate this trend, the proteolysis reaction was prolonged to 4 h (Figure 4), with hemoglobin as the substrate at 37 °C. We used the same amount of ficin in this reaction; the amount of overloaded biocatalyst was eight times greater than the amount of moderately loaded biocatalyst. 

The figure shows that the activity of the overloaded biocatalyst per mg of ficin was slightly lower than that of the moderately loaded biocatalyst. This may be due to the diffusional limitations of the substrate or the existence of steric problems generated by the proximity of other ficin molecules in cases in which the enzyme orientation is not perfect. In any case, the differences in the reaction courses were not very important. However, it is clear that while the reaction rate was only slightly affected by the aldehyde dextran modification with the overloaded biocatalyst, this effect was much greater with the moderately loaded biocatalyst, confirming the results above [83,84].

### 2.3. Effect of the Enzyme Loading and Aldehyde Dextran Modification on the Stability of Immobilized Ficin Biocatalyst

**Figure 5:**
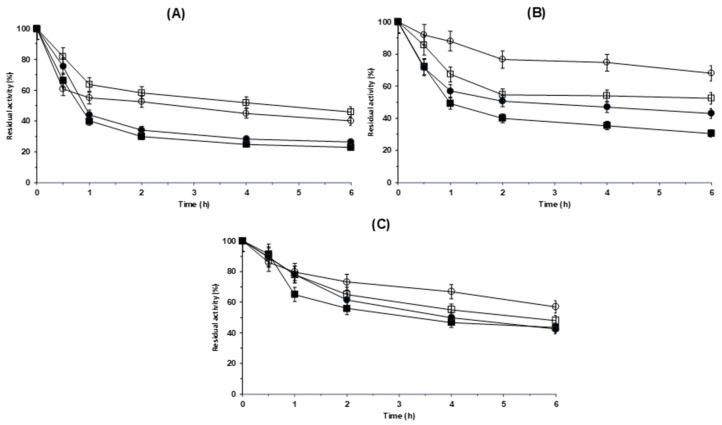
Inactivation courses of different ficin biocatalysts at pH 5 (**A**), pH 7 (**B**) and pH 9 (**C**). The temperature of inactivation was 64 °C. Additional details are available in the Materials and Methods section. Full squares: moderately loaded glyoxyl–ficin; empty squares: moderately loaded glyoxyl–ficin–dextran at 1 mg/mL; full circles: overloaded glyoxyl–ficin; empty circles: overloaded glyoxyl–ficin–dextran.

Finally, we analyzed the stabilities of the different ficin biocatalysts at pH 5, 7 and 9. Figure 5 shows the results. At pH 5, the stabilities of both unmodified biocatalysts were almost identical; at pH 7, the overloaded biocatalyst was more stable than the moderately loaded biocatalyst, and this effect was lower at pH 9. This moderately higher stability of the overloaded biocatalyst can be explained by the fact that with the overloaded biocatalyst, the diffusional limitations of the substrate render the enzyme molecules in the inner part of the biocatalyst unable to initially express full activity due to the lack of substrate [112,113]. Thus, when the biocatalyst activity decreases, the active molecules located in this inner position can express activity, resulting in an apparent enzyme stabilization. However, this should occur at all pH values, but we observed that the effect of the enzyme loading was greater when the pH increased. The possibility of the promotion of enzyme–enzyme interactions with the overloaded biocatalysts could explain, at least in part, the effect of the enzyme loading on the enzyme stability [114,115], and this effect may depend on the inactivation conditions [116,117]. Furthermore, as catalytic Cys oxidation plays an important role in the stability of the ficin [110], perhaps a higher concentration of ficin will also produce an increase in the Cys concentration and thus produce an apparent stabilization of the immobilized enzymes. In any case, the differences in the stabilities of the overloaded and moderately loaded biocatalyst were not very important.

The aldehyde dextran modification had a significant effect on the stability of immobilized ficin, and this effect was more significant for the overloaded biocatalyst. The stabilization promoted by chemical modification was higher at pH 7, mainly for the overloaded biocatalyst. At pH 5, this effect was clear, but the differences between lowly and highly loaded modified biocatalysts were not very relevant, and this stabilization effect by the modification became even smaller at pH 9. The stabilizing effect of the aldehyde dextran modification may be due to the promotion of intra- and intermolecular crosslinking caused by these multifunctional molecules. Although the lack of rigidity of aldehyde dextran may not have a significant stabilization effect, it has been used to stabilize some enzymes [118]. The greater stabilization observed with the overloaded biocatalyst may be explained by the fact that the intermolecular crosslinks in this biocatalyst were favored, and their effects coupled to those of the intramolecular crosslinks, while in the case of the moderately loaded biocatalyst, aldehyde dextran mainly produced intramolecular crosslinks [119]. 

The biocatalysts could be reused to hydrolyze casein for six consecutive cycles of 4 h of proteolysis at 37 °C without alterations in hydrolysis performance. Using for a seventh hydrolysis cycle the biocatalysts, this time using hemoglobin as substrate, the severe reduction in its hydrolysis by the modification of the moderately loaded biocatalyst was maintained, while the unmodified biocatalysts exhibited the same activity.

## 3. Materials and Methods

### 3.1. Materials

Agarose beads 4% BCL support was purchased from Agarose Bead Technologies (ABT) (Alcobendas, Spain). 2,2′-dipyridyldisulfide (2PDS) and hemoglobin were purchased from Thermo Fisher (Kandel, Germany). Sodium periodate, hemoglobin, ethylenediaminetetraacetic acid (EDTA), casein, glycidol, sodium borohydride, 2-mercaptoethanol, benzoyl-arginine-p-nitroanilide (BAPNA) and cysteine were supplied by Sigma-Aldrich (St. Louis, MO, USA). All other reagents were of analytical grade. Glyoxyl agarose beads support was prepared following the protocols and directions of previous works [120].

### 3.2. Methods 

#### 3.2.1. Ficin Preparation

After taking samples of latex from *Ficus carica* L. (stored at 4 °C), these samples were centrifuged (10,000× *g* for 15 min at 4 °C), and the supernatants were retained and combined into a single sample and later divided into 5 mL tubes. The crude ficin extract had a protein concentration of 2 mg/mL. It was stored at −20 °C until it was used. The Bradford method (employing BSA as reference) was utilized to quantify the concentration of protein [121].

#### 3.2.2. Preparation of Aldehyde Dextran

After dissolving 3.33 g of dextran (from *Leucconostoc* spp.) (MW 40,000 Da) in 100 mL of distilled water at 25 °C, 8 g of sodium periodate was incorporated to achieve full dextran oxidation [122]. After 3 h of continuous stirring, the solution was dialyzed against 50 volumes of distilled water, and 6 water changes were performed (2 h, 4 h, 6 h, 8 h, 18 h and 24 h). The resulting aldehyde dextran was stored at 4 °C.

#### 3.2.3. Enzymatic Assays 

The activity of the different ficin biocatalysts was measured using three different substrates: BAPNA (a synthetic and small substrate), casein (small protein) and hemoglobin (large protein). The use of BAPNA is described in [109]. A total of 43.5 mg of BAPNA was added to 1.0 mL of dimethyl sulfoxide, and after it dissolved, the solution was added to 99 mL of 0.1 M sodium phosphate (pH 7.0) containing 5 mM EDTA and 5 mM cysteine. The standard enzyme activity assay was carried out by determining the increase in absorbance at 405 nm, which was enabled by the release of *p*-nitroaniline (*ε* was 8800). To start the reaction, 200 µL of suspension or solution of biocatalyst was added to 1 mL of the substrate solution at 55 °C, and the reaction proceeded for 10 min. Activity is in micromoles of released *p*-nitroaniline per minute. The spectrophotometer that was utilized was a Jasco V 730 (Madrid, Spain), with magnetic stirring and temperature control. 

The Kunitz protocol was used to determine the proteolytic activity (employing casein or hemoglobin) [123], with some small alterations. A solution of 1% (*w*/*v*) protein was prepared in a solution composed of 50 mM sodium phosphate (pH 7.0) that also contained 5 mM cysteine and 5 mM EDTA. A volume of 1 mL of this solution was heated to 37 °C or 55 °C, and then 200 μL of biocatalyst suspension or solution was added; the reaction was maintained under gentle stirring for 15 min. To stop the reaction, 2 mL of 10% trichloroacetic acid (TCA) was added, and then the suspension was centrifuged at 10,000 rpm for 5 min. This treatment induced the precipitation of the non-hydrolyzed protein, but the small peptides produced by the hydrolysis of the proteins remained in solution. The absorbance of this supernatant was determined at 280 nm. To generate a blank, substrate was added after enzyme incubation in 10% TCA, which inactivated the enzyme. One unit of proteolytic activity is defined as the amount of enzyme that increases the absorbance by 0.001 min^−1^ under the given assay conditions. In some instances, the reaction time was prolonged.

#### 3.2.4. Immobilization of Ficin Extract on Glyoxyl Agarose Beads

A mass of 10 g of glyoxyl agarose was added to 100 mL of 1 mg/mL (moderate enzymatic loading) or 9 mg/mL (maximum enzymatic loading) ficin extract solution prepared in 50 mM sodium carbonate at pH 10.05, and this mixture was kept at room temperature for different time periods with continuous stirring [110]. For the final step of immobilization, the suspension was reduced by the addition of solid NaBH_4_ until a concentration of 1 mg/mL was reached. This reaction was carried out at room temperature with gentle agitation. After 30 min, the biocatalyst was thoroughly washed with distilled water and stored at 4 °C [124]. 

#### 3.2.5. Modification of Immobilized Ficin on Glyoxyl Agarose with Aldehyde Dextran

Immobilized ficin was suspended at room temperature in dextran aldehyde solution at pH 8 (1 g/10 mL) and constantly agitated for 48 h. Then, the solution was reduced with sodium borohydride, filtered and washed with an excess of distilled water before storage at 4 °C.

#### 3.2.6. Thermal Inactivation of the Different Ficin Preparations

Glyoxyl–ficin and hemoglobin–glyoxyl–ficin–aldehyde dextran biocatalysts were inactivated by incubation at 64 °C in 50 mM of different buffers (sodium phosphate at pH 7, sodium acetate at pH 5 or carbonate at pH 9). At different times throughout the inactivation process, samples were taken, and the activity of the biocatalysts was determined using casein as the substrate.

#### 3.2.7. Use of Glyoxyl–Ficin–Dextran Biocatalysts for Casein and Hemoglobin Hydrolysis

The resulting biocatalysts were used for casein and hemoglobin hydrolysis at pH 7 and 37 °C or 55 °C. Proteolysis reactions were carried out in a reactor at a controlled temperature with constant agitation. A mass of 0.5 g of biocatalyst was added to 10 mL of 1% (*w*/*v*) of the corresponding protein, which was prepared in 50 mM sodium phosphate buffer containing 5 mM cysteine and 5 mM EDTA at pH 7. At defined times, samples were obtained, and the absorbance was determined as described in the above section.

## 4. Conclusions

The modification of immobilized ficin with aldehyde dextran can have diverse effects depending on the enzyme loading. Greater stabilization effects as a consequence of this modification were found with the overloaded biocatalysts, but the most interesting observation was the effect of this modification on the proteolytic activity: while the moderately loaded biocatalyst had a drastically decreased proteolytic activity against hemoglobin, for the overloaded biocatalyst, this effect was not very relevant. Thus, if this strategy is used to alter the enzyme specificity for different proteins (generated by differences in size or just by altering the protein substrate specificity), moderately loaded biocatalysts will provide better results. If the objective of the modification is to increase the enzyme stabilization without losing the proteolytic capacity of the enzyme, the overloaded biocatalyst may be a good solution. Performing experiments with a variety of proteins that are good substrates of ficin and that have different sequences and sizes can help to advance the understanding of the results of this study (e.g., if the main effect of the dextran modification is a change in the substrate size specificity or just a change in the sequence specificity of the immobilized enzyme).

## Figures and Tables

**Table 1:** Activities of different glyoxyl–ficin biocatalysts against casein and hemoglobin at 37 °C and 55 °C, expressed as a percentage (%). Additional details can be found in the Materials and Methods section. The activity of the unmodified biocatalyst was 100%. At 37 °C, the activity was 70% of that at 55 °C for the glyoxyl–ficin preparation (HG: hemoglobin).

	Activity, (%)				
Substrate	T, (°C)	Moderately Loaded Glyoxyl–Ficin	Moderately Loaded Glyoxyl–Ficin–Dextran	Overloaded Glyoxyl–Ficin	Overloaded Glyoxyl–Ficin– Dextran
**CASEIN**	37	100	71 ± 4	100	93 ± 4
	55	100	9 ± 1	100	90 ± 3
**HG**	37	100	81 ± 3	100	98 ± 5
	55	100	12 ± 4	100	95 ± 4

## Data Availability

The original contributions presented in the study are included in the article, further inquiries can be directed to the corresponding authors.
